# Concordance between FVC and FEV6 for identifying chronic airflow obstruction and spirometric restriction in the Burden of Obstructive Lung Disease (BOLD) study

**DOI:** 10.1136/bmjresp-2024-002355

**Published:** 2025-07-13

**Authors:** Ben Knox-Brown, James Potts, Frits M E Franssen, Rune Nielsen, Meriam Denguezli, Anders Ørskov Rotevatn, Sanjay K Juvekar, Hamid Hacene Cherkaski, Michael Studnicka, Karl Peter Sylvester, Kevin Mortimer, Eric D Bateman, Christer Janson, Andrei Malinovschi, Terence Seemungal, Parvaiz Koul, David Mannino, Padukudru Anand Mahesh, Rain Jogi, Filip Mejza, Mohammed Al Ghobain, Stefanni Nonna M Paraguas, Tobias Welte, Emiel Wouters, Thorarinn Gislason, Imed Harrabi, Hermínia Dias, Daniel O Obaseki, Ali Kocabas, Cristina Barbara, Joao Cardoso, Dhiraj Agarwal, Asaad Ahmed Nafees, Fatima Rodrigues, Vanessa Garcia-Larsen, Gregory E Erhabor, Li-Cher Loh, Andre F S Amaral, on behalf of the BOLD collaborative research group, Hasan Hafizi

**Affiliations:** 1 Imperial College London National Heart and Lung Institute, London, UK; 2 Cambridge Respiratory Physiology, Royal Papworth and Cambridge University Hospitals NHS FT, Cambridge, UK; 3 National Heart and Lung Institute, Imperial College London, London, UK; 4 Department of Respiratory Medicine, Maastricht University Medical Centre, Maastricht, The Netherlands; 5 Department of Thoracic Medicine, Haukeland University Hospital, Bergen, Norway; 6 Faculté de Médecine de Sousse, Université de Sousse, Sousse, Tunisia; 7 Department of Clinical Science, University of Bergen, Bergen, Norway; 8 Vadu Rural Health Program, KEM Hospital Pune Research Centre, Pune, Maharashtra, India; 9 Dr D Y Patil Medical College Hospital and Research Centre, Pune, Maharashtra, India; 10 Department of Pneumology, University Badji Mokhtar of Annaba, Annaba, Algeria; 11 Department Respiratory Disease, Paracelsus Medical University Salzburg, Salzburg, Austria; 12 University of Cambridge, Cambridge, UK; 13 Liverpool University Hospitals NHS Foundation Trust, Liverpool, UK; 14 Department of Medicine, University of Cape Town, Rondebosch, South Africa; 15 Dep of Medical Sciences: Respiratory, Allergy and Sleep Research, Uppsala University, Uppsala, Sweden; 16 The University of the West Indies St Augustine Campus, St Augustine, Trinidad and Tobago; 17 Department of Pulmonary Medicine, Sher-i-Kashmir Institute of Medical Sciences, Srinagar, Jammu and Kashmir, India; 18 University of Kentucky, Lexington, Kentucky, USA; 19 COPD Foundation, Miami, Florida, USA; 20 Department of Respiratory Medicine, JSS Medical College, Mysuru, Karnataka, India; 21 Lung Clinic, Tartu University Hospital, Tartu, Estonia; 22 Centre for Evidence Based Medicine, 2nd Department of Internal Medicine, Jagiellonian University Medical College, Krakow, Poland; 23 King Saud bin Abdulaziz University for Health Sciences, Riyadh, Saudi Arabia; 24 King Abdullah International Medical Research Center, Riyadh, Saudi Arabia; 25 Philippine College of Chest Physicians, Manila, Philippines; 26 Department of Pneumology, Hannover Medical School, Hannover, Germany; 27 Medical Faculty, Sigmund Freud Private University Vienna, Vienna, Austria; 28 Maastricht University Medical Centre+, Maastricht, The Netherlands; 29 Faculty of Medicine, University of Iceland, Reykjavik, Iceland; 30 Department of Sleep, Landspitali, Reykjavik, Iceland; 31 Ibn El Jazzar Faculty of Medicine of Sousse, University of Sousse, Sousse, Tunisia; 32 Escola Superior de Tecnologia da Saúde de Lisboa, Lisbon Polytechnic Institute Lisbon School of Health Technology, Lisboa, Portugal; 33 Department of Medicine, Obafemi Awolowo University, Ile-Ife, Nigeria; 34 Faculty of Medicine, The University of British Columbia, Vancouver, British Columbia, Canada; 35 Department of Chest Diseases, Çukuova University, Adana, Turkey; 36 Institute of Environmental Health, University of Lisbon, Lisboa, Portugal; 37 Centro Hospitalar Universitário Lisboa Norte EPE Serviço de Pneumologia, Lisboa, Portugal; 38 Department of Community Health Sciences, The Aga Khan University, Karachi, Pakistan; 39 Department of International Health, Johns Hopkins Bloomberg School of Public Health, Baltimore, Maryland, USA; 40 Obafemi Awolowo University, Ile-Ife, Nigeria; 41 Royal College of Surgeons in Ireland, University College Dublin Malaysia Campus, Penang, Malaysia; 42 NHLI, Imperial College London, London, UK; 43 NIHR Imperial Biomedical Research Centre, London, UK

**Keywords:** Respiratory Function Test, Respiratory Measurement, Lung Physiology, Sensitivity and Specificity, Clinical Epidemiology

## Abstract

**Introduction:**

We investigated whether the forced expiratory volume in 6 s (FEV_6_) can be used as a surrogate for the forced vital capacity (FVC).

**Methods:**

The Burden of Obstructive Lung Disease is a multinational cohort study. At baseline, data were collected from adults, aged 40 years or older, from 41 sites across 34 countries. Participants from 18 sites were followed-up after a median of 8.3 years. Participants who completed the study core questionnaire and had acceptable post-bronchodilator spirometry were included. We performed receiver operating characteristic analyses to measure the ability of FEV_1_/FEV_6_ less than the lower limit of normal (LLN) to correctly classify FEV_1_/FVC less than the LLN, and FEV_6_ less than the LLN to correctly classify FVC less than the LLN. We used multilevel regression analyses to assess the association of discordant measurements with respiratory symptoms, quality of life and lung function decline.

**Results:**

At baseline, 28 604 participants were included. 53% were female (15 060). 10% (2876) had chronic airflow obstruction for FEV_1_/FVC, compared with 9% (2704) for FEV_1_/FEV_6_. 37% (10 637) had spirometric restriction for FVC, compared with 35% (9978) for FEV_6_. The FEV_1_/FEV_6_ had excellent accuracy in identifying FEV_1_/FVC less than the LLN (area under the curve (AUC): 0.90, 95% CI, 0.89 to 0.91, κ coefficient 0.82). The FEV_6_ also had excellent agreement in identifying FVC less than the LLN (AUC: 0.95, 95% CI, 0.94 to 0.95, κ coefficient 0.90). Discordant reductions in FEV_1_/FEV_6_ (1%, 345) and FEV_6_ (1%, 309) were associated with greater odds of having respiratory symptoms and a lower physical quality of life. 3870 participants were followed up. Those with discordant reductions in FEV_1_/FEV_6_ and FEV_6_ were more likely to have chronic airflow obstruction and spirometric restriction at follow-up.

**Conclusions:**

There is strong agreement between the FVC and FEV_6_ in the identification of chronic airflow obstruction and spirometric restriction.

**WHAT IS ALREADY KNOWN ON THIS TOPIC**

- Previous studies in clinical populations across a limited number of countries have shown good agreement between forced vital capacity (FVC) and forced expiratory volume in 6 s (FEV_6_) in the identification of chronic airflow obstruction and spirometric restriction.

**WHAT THIS STUDY ADDS**

- Across multiple word regions, there is strong agreement between the FVC and FEV_6_ in the identification of chronic airflow obstruction and spirometric restriction. Discordance was seen in 1% of measurements and was associated with increased respiratory symptoms, lower quality of life and lung function decline.

**HOW THIS STUDY MIGHT AFFECT RESEARCH, PRACTICE OR POLICY**

- The FEV_6_ requires less effort and is easier to reproduce than the FVC. We provide evidence that it can be used to accurately identify chronic airflow obstruction and spirometric restriction. This may be particularly important for home spirometry or low resource settings, where cheaper spirometry devices are used.

## Introduction

 Spirometry is an important component in the diagnosis and management of both obstructive and restrictive lung abnormalities.^1^,^3^ These are typically identified using the forced expiratory volume in 1 s as a ratio of the forced vital capacity (FEV_1_/FVC) and the FVC, respectively. However, the ability to correctly classify these spirometric conditions is dependent on the accuracy of the measurements taken.

Spirometry is volitional, with one of the most common errors being premature termination of the forced expiratory manoeuvre. This can be caused by insufficient effort, discomfort, cough or technician error.^4^ Early termination results in the underestimation of the FVC and overestimation of the FEV_1_/FVC ratio. As a result, there has been interest in the forced expiratory volume in 6 s (FEV_6_) as a surrogate for the FVC.^5^,^15^ The rationale being that the FEV_6_ can provide the same information as the FVC but requires less effort and is easier to reproduce.^16^

Studies generally show very good agreement between FVC and FEV_6_ in the diagnosis of airflow obstruction and spirometric restriction.^5^,^15^ Despite this, most report a degree of discordance, ranging from 1% to 7% of measurements.^14 15^ Bhatt and colleagues^7^ showed, using data from the Chronic Obstructive Pulmonary Disease Gene (COPDGene) study, that participants with a reduced FEV_1_/FEV_6_ ratio (<0.73) had evidence of airways disease on CT imaging, despite having a normal FEV_1_/FVC ratio. However, most studies that have examined concordance are hospital-based^513^,^16^ or in ever smokers.^7 9 10^ This makes it difficult to infer whether similar agreement would be seen across different global populations.

To our knowledge, no study has attempted to investigate the agreement between the FVC and FEV_6_ in the classification of chronic airflow obstruction and spirometric restriction, across multiple world regions. Furthermore, before the FEV_6_ can be integrated into clinical decision-making, thorough investigation of factors associated with discordant measurements is required, including whether discordance reflects a genuine physiological abnormality. We aimed to use data from multiple world regions to assess the degree of agreement between these parameters, examine the factors associated with discordance and identify whether individuals with discordant measurements have accelerated lung function decline over time.

## Methods

### Study design and participants

The Burden of Obstructive Lung Disease (BOLD) study is a multinational cohort study, with two phases of data collection. The protocols for each phase have been published previously.^17 18^ At baseline, non-institutionalised adults aged 40 years and older were recruited from 41 municipalities across 34 countries, where populations were larger than 150 000 people. Site-specific sampling strategies were employed to randomly recruit representative samples of the populations studied. Participants from 18 sites, 14 from low- and middle-income countries and 4 from high-income countries in Northern Europe were then followed up. For the cross-sectional analyses, data were included if the participant had completed the core study questionnaire and had acceptable post-bronchodilator spirometry at baseline, according to predefined quality criteria.^19^ We focused on post-bronchodilator spirometry to limit the impact of reversible airways disease on the classification of spirometric restriction. For the longitudinal analyses, data were included if the participant had completed the core study questionnaire and had good quality spirometry at both baseline and follow-up. Participants were excluded if they had a contraindication for lung function testing.

### Procedures

Information on respiratory symptoms, health status and exposures were collected by trained field workers who administered standardised questionnaires translated into the local language. The FEV_1_, FVC and FEV_6_ were measured using the EasyOne Spirometer (ndd Medizintechnik, Zurich, Switzerland) before and 15 min after administration of 200 µg of inhaled salbutamol. Spirograms were centrally reviewed and assigned a quality score based on acceptability and reproducibility criteria.^19^ Only tests with back-extrapolated volume <150 mL, peak expiratory flow time <120 ms, lasting ≥6 s or with end-of-time volume <40 mL, no artefact affecting the FEV_1_ or FVC and with the two best blows within 200 mL of each other were used. Sex was self-reported by study participants, with male and female options. As the reference standards, we defined chronic airflow obstruction if the post-bronchodilator FEV_1_/FVC ratio was less than the lower limit of normal (LLN) and spirometric restriction if the FVC was less than the LLN. As the comparators, we used the post-bronchodilator FEV_1_/FEV_6_ ratio and FEV_6_ to define chronic airflow obstruction and spirometric restriction if a result was less than the LLN. Based on the agreement between parameters, we defined four different discordant groups: (1) FEV_1_/FVC ratio less than the LLN with a normal FEV_1_/FEV_6_ ratio; (2) FEV_1_/FEV_6_ ratio less than the LLN with a normal FEV_1_/FVC ratio; (3) FVC less than the LLN with a normal FEV_6_; and (4) FEV_6_ less than the LLN with a normal FVC. To calculate the LLN, we used sex-specific coefficients for age and height from reference equations for European Americans in the third US National Health and Nutrition Examination Survey (NHANES).^20^

We investigated factors associated with discordance, including: age (years); body mass index (BMI) (kg/m²); pack-years of smoking (number of cigarettes smoked per day divided by 20 and multiplied by years of smoking); smoking status, categorised as never, former and current; dyspnoea, categorised as minimal/no breathlessness (0–1 on the modified Medical Research Council mMRC dyspnoea scale) and significant breathlessness (≥2 on the mMRC dyspnoea scale); chronic cough, chronic phlegm and wheeze, categorised as yes/no by responses to the following questions: (1) ‘do you cough on most days for as much as 3 months each year?’; (2) ‘do you bring up phlegm on most days for as much 3 months each year?’; and (3) ‘have you had wheezing or whistling in the chest at any time in the last 12 months?’; and physical and mental quality of life (QoL), assessed using the 12-item short form health survey (SF-12), where scores range from 0 to 100, with a score of 100 indicating the best QoL.^21^

### Statistical analysis

We estimated the prevalence of chronic airflow obstruction and spirometric restriction for each definition. Receiver operating characteristic analyses were performed to measure the ability of FEV_1_/FEV_6_ ratio and FEV_6_ to correctly classify the presence of chronic airflow obstruction and spirometric restriction. We evaluated the concordance using Cohen’s κ coefficient.^22^ We stratified these analyses by sex and WHO region to investigate any effect modification and further performed secondary analyses using an alternative definition of spirometric restriction, where participants with an FEV_1_/FVC or FEV_1_/FEV_6_ ratio less than the LLN were excluded. We also repeated these analyses using pre-bronchodilator measurements to check that the accuracy of classification was similar to post-bronchodilator measurements. We used multilevel (mixed effects) logistic regression analyses to assess the association of discordance with respiratory symptoms and multilevel linear regression to assess the association of discordance with QoL. We fitted the multilevel models with a random intercept to account for clustering by study site and a random slope to average the association of respiratory symptoms and QoL across sites. We adjusted for sex, age, BMI, smoking status, and smoking pack-years. Analyses of baseline data were corrected for sampling weights.

At follow-up, to estimate the association between having discordant reductions in FEV_1_/FEV_6_ ratio and FEV_6_ at baseline and progression to chronic airflow obstruction and spirometric restriction at follow-up, we performed multilevel logistic regression analyses. We also used multilevel linear regression to estimate the association between discordant reductions in FEV_1_/FEV_6_ ratio and FEV_6_ and post-bronchodilator FEV_1_/FVC ratio and FVC as continuous measures. We adjusted for sex, age, BMI, smoking status, follow-up time and pack-years of smoking. Analysis of follow-up data was conducted using inverse probability weights^23^ to account for missing data. All analyses were performed using Stata V.17 and results considered significant if the p value was below 0.05.

### Patient and public involvement

Patients or the public were not involved in the design, or conduct, or reporting, or dissemination plans of our research.

## Results

At baseline, 36 618 participants were recruited between 2 January 2003 and 26 December 2016. Data was collected at recruitment. A total of 28 604 participants who had acceptable post-bronchodilator spirometry and completed the core study questionnaire were included in the cross-sectional analyses.

**Figure 1:**
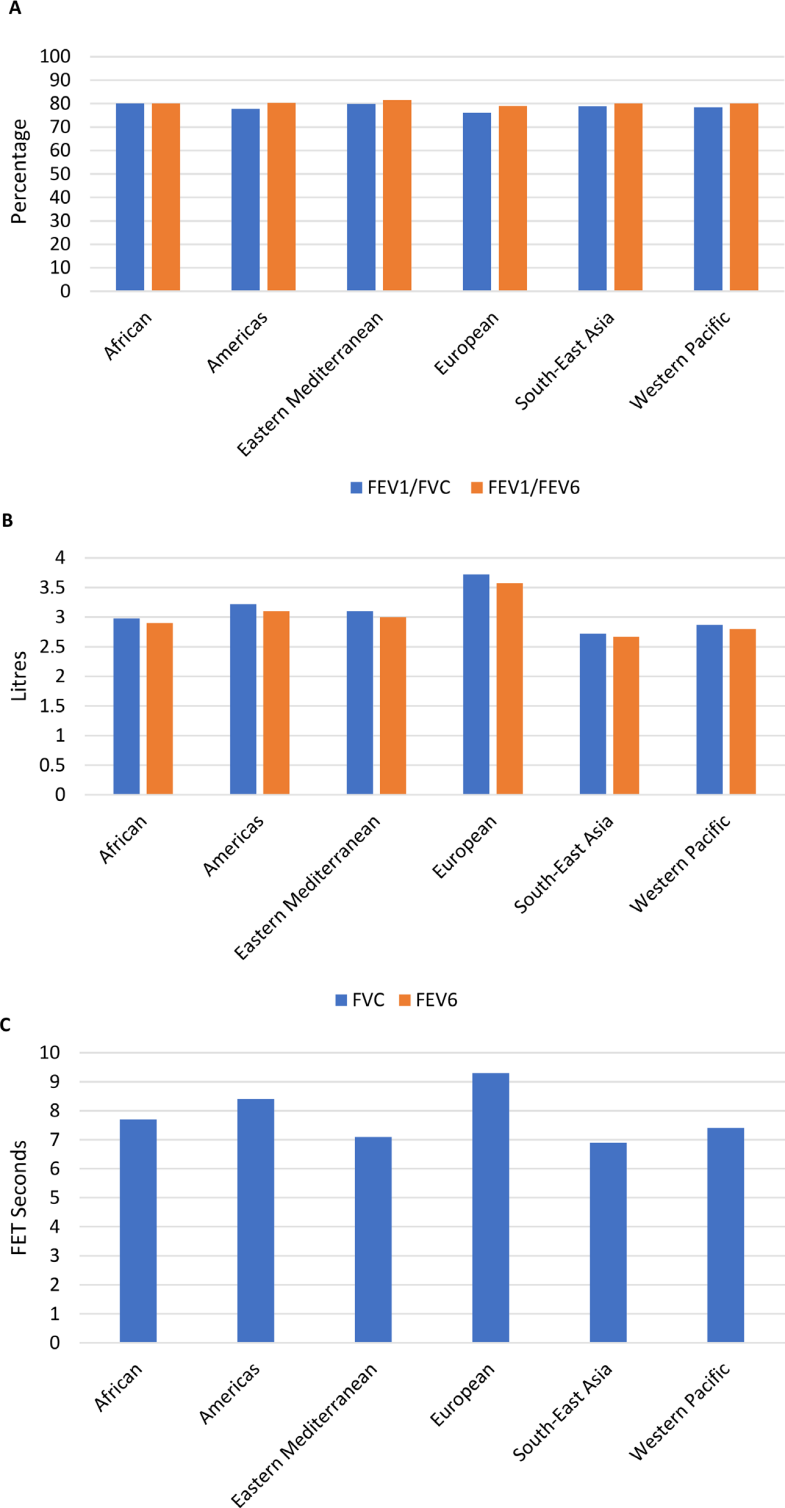
(**A**) Comparison of mean FEV_1_/FVC ratio and FEV_1_/FEV6 ratio across WHO regions. (**B**) Comparison of mean FVC and FEV_6_ across WHO regions. (**C**) Comparison of median forced expiratory time across WHO regions. FET, forced expiratory time; FEV_1_, forced expiratory volume in 1 s; FEV_6_, forced expiratory volume in 6 s; FVC, forced vital capacity.

The characteristics of study participants are displayed in online supplemental eTable 1. There were slightly more females than males (15 060 vs 13 544), mean age ranged from 46.7 years to 63.4 years across sites. The proportion of ever smokers ranged from 2% (13 of 694) in Sémé-Kpodji, Benin, to 68% (570 of 843) in Uitsig and Ravensmead, South Africa. Mean FEV_1_/FVC ratio was lowest in the European region (76.1%) and highest in the African region (80.0%), while mean FEV_1_/FEV_6_ ratio was lowest in the European region (79.0%) and highest in the Eastern Mediterranean region (81.5%) (figure 1a). Mean FVC and FEV_6_ were lowest in the South-East Asia (2.72 L and 2.67 L, respectively) and highest in the European region (3.72 L and 3.57 L, respectively) (figure 1b). Median forced expiratory time was shortest in South-East Asia (6.9 s) and longest in the European region (9.3 s) (figure 1c).

**Figure 2:**
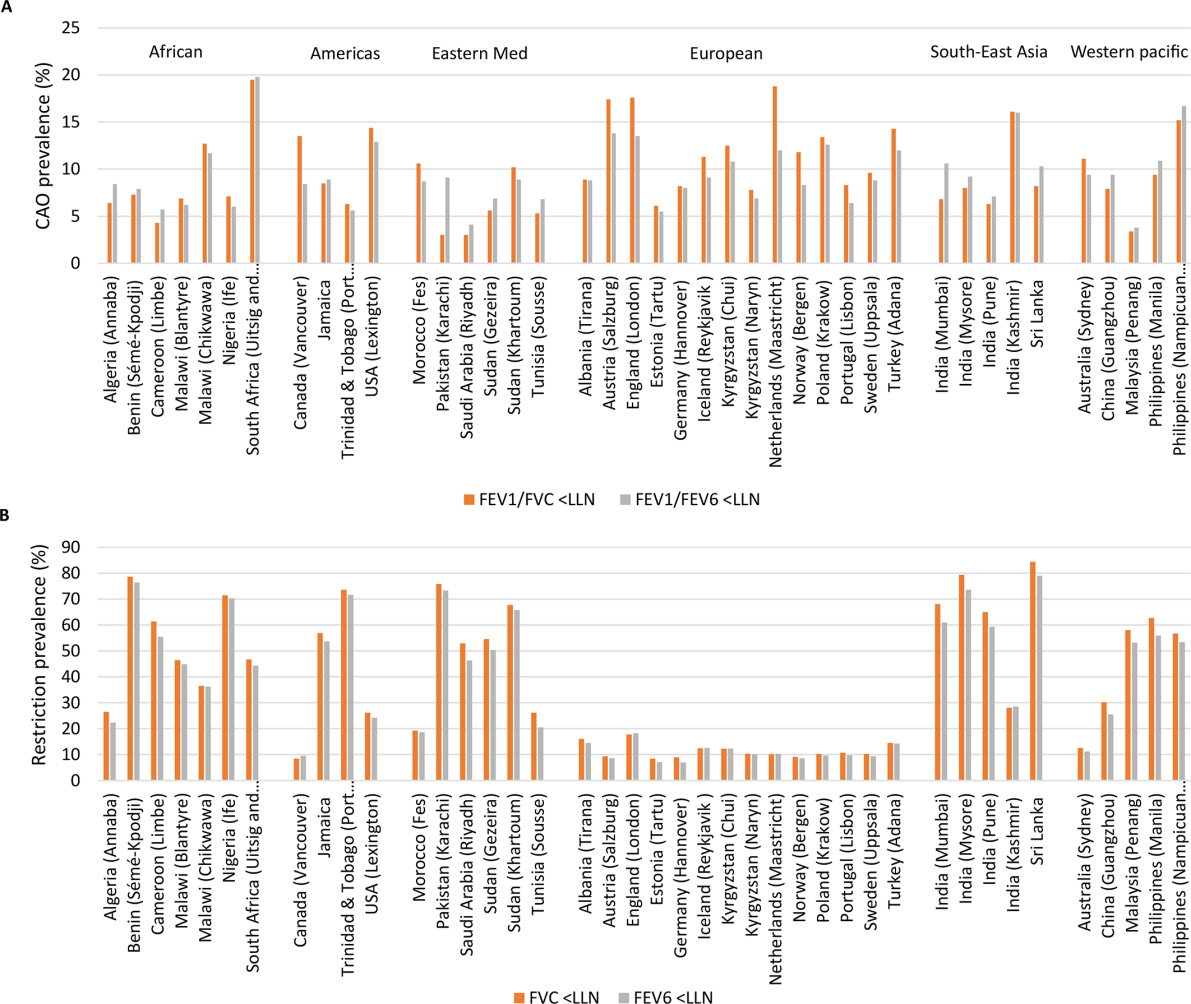
(**A**) Prevalence of chronic airflow obstruction defined using FEV_1_/FVC and FEV_1_/FEV_6_ for each BOLD study site. (**B**) Prevalence of spirometric restriction defined using FVC and FEV_6_ for each BOLD centre. BOLD, Burden of Obstructive Lung Disease study; CAO, chronic airflow obstruction; FEV_1_, forced expiratory volume in 1 s; FEV_6_, forced expiratory volume in 6 s; FVC, forced vital capacity; LLN, lower limit of normal.

Of the total population, 10% (2876 of 28 604) had chronic airflow obstruction for FEV_1_/FVC ratio less than the LLN, compared with 9% (2704 of 28 604) for FEV_1_/FEV_6_ ratio less than the LLN. Characteristics were similar among those with chronic airflow obstruction regardless of definition (table 1). By BOLD centre, the prevalence of chronic airflow obstruction ranged from 3% (21 of 700) in Riyadh, Saudi Arabia, to 19.5% (164 of 843) in Uitsig and Ravensmead, South Africa, for FEV_1_/FVC ratio less than the LLN. For FEV_1_/FEV_6_ ratio less than the LLN, the prevalence of chronic airflow obstruction ranged from 3.8% (25 of 663) in Penang, Malaysia, to 19.8% (167 of 843) in Uitsig and Ravensmead, South Africa (figure 2a). 37% (10 637 of 28 604) of the study population had spirometric restriction for FVC less than the LLN, compared with 35% (9978 of 28 604) for FEV_6_ less than the LLN. Characteristics were similar among those with spirometric restriction regardless of definition (table 1). By BOLD centre, the prevalence of spirometric restriction ranged from 8.5% (70 of 826) in Vancouver, Canada, and (52 of 613) Tartu, Estonia, to 84.4% (863 of 1023) in Sri Lanka. For FEV_6_ less than the LLN, prevalence of spirometric restriction ranged from 7.0% (48 of 683) in Hannover, Germany, to 79.0% (808 of 1023) in Sri Lanka (figure 2b).

**Table 1:** Characteristics of those with chronic airflow obstruction defined using FEV_1_/FVC and FEV_1_/FEV_6_ and spirometric restriction defined using FVC and FEV_6_

n*=*28 604	Chronic airflow obstruction		Spirometric restriction	
	FEV_1_/FVC(<LLN)	FEV_1_/FEV_6_(<LLN)	FVC(<LLN)	FEV_6_(<LLN)
Total, n (%)	2876 (10)	2704 (9)	10 637 (37)	9978 (35)
Female sex, n (%)	1326 (46)	1212 (45)	5763 (54)	5459 (55)
Age years, mean (SD)	60 (11.9)	59.4 (11.9)	52.6 (9.6)	52.4 (9.6)
BMI kg/m^2^, mean (SD)	25.3 (7.7)	24.9 (7.9)	26.6 (6.2)	26.5 (6.2)
Ever smoke, n (%)	1671 (58)	1518 (56)	3101 (29)	2935 (29)
FEV_1_, L, mean (SD)	1.9 (0.8)	1.8 (0.7)	2.0 (0.6)	2.0 (0.6)
FEV_6_, L, mean (SD)	2.9 (1.0)	2.8 (1.0)	2.5 (0.7)	2.5 (0.7)
FVC, L, mean (SD)	3.2 (1.1)	3.0 (1.1)	2.6 (0.7)	2.6 (0.7)
FEV_1_/FVC, mean (SD)	59.0 (9.2)	59.1 (9.6)	78.8 (9.5)	77.9 (10.5)
FEV_1_/FEV_6_, mean (SD)	64.8 (8.3)	63.9 (7.8)	80.4 (8.2)	79.8 (8.8)
Dyspnoea, n (%)	1038 (36)	1010 (38)	2657 (25)	2537 (25)
FET, seconds, median (IQR)	11.1 (9.0–14.4)	9.8 (8.1–12.9)	7.1 (6.4–8.5)	7.2 (6.5–8.8)
Chronic cough, n (%)	507 (18)	477 (18)	747 (7)	754 (8)
Chronic phlegm, n (%)	498 (17)	486 (18)	772 (2)	754 (8)
Wheeze, n (%)	1090 (38)	1042 (39)	1884 (18)	1844 (19)

Categorical variables summarised as number with (%). Continuous variables summarised as mean with SD.

Spirometric condition identified if a result is less than the LLN. To calculate the LLN, we used sex-specific coefficients for age and height from reference equations for European Americans in the third US National Health and Nutrition Examination Survey.

BMI, body mass index; FET, forced expiratory time; FEV_1_, forced expiratory volume in 1 s; FEV_6_, forced expiratory volume in 6 s; FVC, forced vital capacity; LLN, lower limit of normal.

Table 2 summarises the agreement between parameters in the identification of chronic airflow obstruction and spirometric restriction. Overall, the FEV_1_/FEV_6_ ratio less than the LLN had good accuracy in identifying chronic airflow obstruction defined as FEV_1_/FVC ratio less than the LLN, with an area under the curve (AUC) of 0.90 (95% CI, 0.89 to 0.91) and a κ coefficient of 0.82. Agreement was similarly strong among males and females separately. The level of agreement was the same when pre-bronchodilator measurements were used (online supplemental eTable 2). When stratifying by WHO region, the AUC ranged from 0.86 (95%CI, 0.84 to 0.89) with a κ coefficient of 0.80 in the Americas, to an AUC of 0.96 (95%CI, 0.94 to 0.97) with a κ coefficient of 0.86 in South-East Asia. Overall, the FEV_6_ less than the LLN had good agreement in identifying spirometric restriction defined as FVC less than the LLN, with an AUC of 0.95 (95% CI, 0.94 to 0.95) and κ coefficient of 0.90. There was minimal difference in agreement between males and females. When stratifying by WHO region, the AUC ranged from 0.94 (95% CI, 0.94 to 0.95) with a κ coefficient of 0.89 in the Western Pacific, to an AUC of 0.96 (95% CI, 0.95 to 0.96) with a κ coefficient of 0.91 in the Americas. Results were similar when defining spirometric restriction in those with an FVC less than the LLN and normal FEV_1_/FVC ratio and FEV_6_ less than the LLN with normal FEV_1_/FEV_6_ ratio (online supplemental eTable 3).

**Table 2:** Ability of FEV_1_/FEV_6_ and FEV_6_ less than the LLN to identify chronic airflow obstruction and spirometric restriction defined using FEV_1_/FVC and FVC less than the LLN

	n	Level of agreement %	Sensitivity%	Specificity%	AUC(95% CI)	Kappa coefficient (SE)
**Chronic airflow obstruction**						
Overall	28 604	97.00	82.02	98.66	0.90 (0.89 to 0.91)	0.82 (0.01)
Male	13 544	96.75	83.94	98.42	0.91 (0.90 to 0.92)	0.84 (0.01)
Female	15 060	97.20	79.79	98.88	0.89 (0.88 to 0.90)	0.82 (0.01)
WHO region						
African	4430	97.27	87.76	98.32	0.93 (0.91 to 0.95)	0.85 (0.02)
Americas	3004	96.60	74.75	99.04	0.86 (0.84 to 0.89)	0.80 (0.02)
Eastern Mediterranean	3833	97.81	86.18	98.71	0.92 (0.90 to 0.94)	0.84 (0.02)
European	10 442	96.29	75.28	99.10	0.87 (0.86 to 0.88)	0.81 (0.01)
South-East Asia	3603	97.47	94.21	97.80	0.96 (0.94 to 0.97)	0.86 (0.02)
Western Pacific	3292	97.66	91.36	98.29	0.95 (0.93 to 0.96)	0.86 (0.02)
**Spirometric restriction**						
Overall	28 604	95.54	90.90	98.28	0.95 (0.94 to 0.95)	0.90 (0.01)
Male	13 544	94.78	89.11	97.97	0.94 (0.93 to 0.94)	0.88 (0.01)
Female	15 060	96.22	92.42	98.57	0.96 (0.95 to 0.96)	0.92 (0.01)
WHO region						
African	4430	94.56	92.39	96.96	0.95 (0.94 to 0.95)	0.89 (0.02)
Americas	3004	95.71	93.75	97.29	0.96 (0.95 to 0.96)	0.91 (0.02)
Eastern Mediterranean	3833	94.65	90.62	98.35	0.95 (0.94 to 0.95)	0.89 (0.02)
European	10 442	97.01	84.60	98.64	0.92 (0.91 to 0.93)	0.85 (0.01)
South-East Asia	3603	94.25	92.16	98.11	0.95 (0.94 to 0.96)	0.88 (0.02)
Western Pacific	3292	94.44	89.58	99.00	0.94 (0.94 to 0.95)	0.89 (0.02)

Level of agreement classified according to Cohen 1960,^22^ 0.01–0.20=none to minimal, 0.21–0.40=slight, 0.41–0.60=moderate, 0.61–0.80=substantial, 0.81–1.0=almost perfect.

Spirometric condition identified if a result is less than the LLN. To calculate the LLN, we used sex-specific coefficients for age and height from reference equations for European Americans in the third US National Health and Nutrition Examination Survey.^20^

AUC, area under the curve; FEV_1_, forced expiratory volume in one second; FEV_6_, forced expiratory volume in 6 seconds; FVC, forced vital capacity; LLN, lower limit of normal.

The prevalence of discordant measurements among those with chronic airflow obstruction was 2% (517 of 28 604) for FEV_1_/FVC ratio less than the LLN when the FEV_1_/FEV_6_ ratio was normal, and 1% (345 of 28 604) for FEV_1_/FEV_6_ ratio less than the LLN when the FEV_1_/FVC ratio was normal. The prevalence of discordant measurements among those with spirometric restriction was 3% (968 of 28 604) for FVC less than the LLN when the FEV_6_ was normal, and 1% (309 of 28 604) for FEV_6_ less than the LLN when the FVC was normal. Table 3 summarises the characteristics of these discordant groups in comparison to those with concordant lung function. Those with discordant chronic airflow obstruction were of a similar age and BMI to those with concordant measurements. However, those who were concordant generally had more airflow obstruction and a greater symptom burden. Likewise, those with discordant spirometric restriction were of a similar age and BMI to those with concordant measurements. However, those with a discordant reduction in FEV_6_ were generally more symptomatic and had lower FEV_1_/FVC and FEV_1_/FEV_6_ ratios.

**Table 3:** Characteristics of those with concordant and discordant spirometry according to spirometric condition

n=28 604	Chronic airflow obstruction			Spirometric restriction		
	FEV_1_/FVC and FEV_1_/FEV_6_<LLN	FEV_1_/FVC<LLN when FEV_1_/FEV_6_≥LLN	FEV_1_/FEV_6_<LLN when FEV_1_/FVC≥LLN	FVC and FEV_6_<LLN	FVC<LLN when FEV_6_≥LLN	FEV_6_<LLN when FVC≥LLN
Total, n (%)	2359 (8)	517 (2)	345 (1)	9669 (34)	968 (3)	309 (1)
Female sex, n (%)	1048 (45)	268 (52)	154 (45)	5326 (55)	437 (45)	133 (43)
Age years, mean (SD)	59.5 (12.0)	59.5 (11.5)	58.3 (11.9)	52.2 (9.5)	56.5 (10.4)	58.0 (11.2)
BMI kg/m^2^, mean (SD)	25.0 (8.2)	26.7 (5.0)	23.9 (5.5)	26.5 (6.2)	26.7 (6.1)	26.0 (5.8)
Ever smoke, n (%)	1364 (58)	306 (59)	153 (44)	2759 (29)	344 (36)	178 (58)
FEV_1_, L, mean (SD)	1.8 (0.7)	2.4 (0.7)	2.0 (0.8)	2.0 (0.6)	2.4 (0.6)	1.9 (0.7)
FEV_6_, L, mean (SD)	2.8 (1.0)	3.3 (1.0)	2.9 (1.1)	2.5 (0.6)	2.9 (0.7)	2.8 (0.7)
FVC, L, mean (SD)	3.0 (1.1)	3.7 (1.1)	3.0 (1.1)	2.5 (0.7)	2.9 (0.7)	3.2 (0.8)
FEV_1_/FVC, mean (SD)	57.6 (9.5)	65.3 (3.7)	68.7 (2.8)	78.5 (9.8)	81.5 (5.7)	59.2 (14.2)
FEV_1_/FEV_6_, mean (SD)	63.0 (7.9)	73.1 (2.6)	70.0 (2.5)	80.2 (8.4)	82.3 (5.4)	66.9 (11.9)
FET, seconds, Median (IQR)	10.3 (8.6–13.4)	9.3 (7.0–13.8)	7.3 (6.5–8.7)	7.2 (6.4–8.6)	6.9 (6.2–7.6)	14.5 (11.2–16.7)
Dyspnoea, n (%)	911 (39)	127 (25)	99 (29)	2434 (25)	223 (23)	103 (33)
Chronic cough, n (%)	447 (19)	60 (12)	30 (9)	705 (7)	42 (4)	49 (16)
Chronic phlegm, n (%)	440 (19)	58 (11)	46 (13)	710 (7)	62 (6)	44 (14)
Wheeze, n (%),	957 (41)	133 (26)	85 (24)	1728 (18)	156 (16)	116 (37)
Physical QoL (SF-12), mean (SD)	44.2 (10.5)	46.8 (9.9)	45.8 (9.3)	46.4 (9.1)	47.6 (9.2)	44.8 (9.9)
Mental QoL (SF-12), mean (SD)	50.0 (10.4)	50.7 (10.0)	50.4 (9.7)	50.2 (10.0)	52.2 (9.7)	51.3 (10.6)

To calculate the LLN, we used sex-specific coefficients for age and height from reference equations for European Americans in the third US National Health and Nutrition Examination Survey.^1^

BMI, body mass index; FET, forced expiratory time; FEV_1_, forced expiratory volume in 1 s; FEV_6_, forced expiratory volume in 6 s; FVC, forced vital capacity; LLN, lower limit of normal; QoL, quality of life assessed using SF-12 questionnaire; SF-12, 12-item short form health survey.

Those with discordant chronic airflow obstruction had significantly higher odds of reporting dyspnoea, chronic cough, chronic phlegm and wheeze than those with normal lung function (table 4). However, the magnitude of the association was smaller than that seen when there was agreement between the FEV_1_/FVC ratio and FEV_1_/FEV_6_ ratio less than the LLN. Those with a discordant reduction in FEV_1_/FEV_6_ ratio had a mean FEV_1_/FVC ratio close to the LLN (mean difference from LLN=1.4%). For discordant spirometric restriction, having an FVC less than the LLN with a normal FEV_6_ was not associated with any respiratory symptom. Whereas having an FEV_6_ less than the LLN with a normal FVC was associated with increased odds of all respiratory symptoms (table 5). Both discordant definitions of chronic airflow obstruction were associated with a lower physical QoL (table 4). However, only having an FEV_1_/FEV_6_ ratio less than the LLN with a normal FEV_1_/FVC ratio was associated with a lower mental QoL. For spirometric restriction, only those with an FEV_6_ less than the LLN and normal FVC had a lower physical QoL (table 5).

**Table 4:** Association of concordant and discordant measurements with respiratory symptoms

	Dyspnoea		Chronic cough		Chronic phlegm		Wheeze	
	OR (95% CI)	P value	OR (95% CI)	P value	OR (95% CI)	P value	OR (95% CI)	P value
Chronic airflow obstruction								
FEV_1_/FVC and FEV_1_/FEV_6_<LLN (n=2359)	2.35 (1.95 to 2.83)	<0.0001	2.65 (2.18 to 3.23)	<0.0001	2.53 (2.05 to 3.11)	<0.0001	4.04 (3.46 to 4.72)	<0.0001
FEV_1_/FVC<LLN with FEV_1_/FEV_6_≥LLN (n=517)	1.54 (1.22 to 1.53)	<0.0001	1.55 (1.16 to 2.06)	0.003	1.59 (1.09 to 2.29)	0.015	1.67 (1.34 to 2.09)	<0.0001
FEV_1_/FEV_6_<LLN with FEV_1_/FVC≥LLN (n=345)	1.50 (1.06 to 2.11)	0.019	1.52 (1.03 to 2.25)	0.035	2.29 (1.65 to 3.21)	<0.0001	2.21 (1.59 to 3.01)	<0.0001
Spirometric restriction								
FVC and FEV_6_<LLN (n=9669)	1.23 (1.09 to 1.39)	0.001	1.25 (1.06 to 1.47)	0.007	1.15 (0.97 to 1.36)	0.098	1.24 (1.11 to 1.38)	<0.0001
FVC<LLN with FEV_6_≥LLN (n=968)	0.97 (0.81 to 1.16)	0.728	0.65 (0.43 to 1.01)	0.051	0.97 (0.74 to 1.29)	0.863	1.07 (0.88 to 1.31)	0.490
FEV_6_<LLN with FVC≥LLN (n=309)	1.95 (1.43 to 2.64)	<0.0001	1.56 (0.92 to 2.61)	0.096	1.66 (1.07 to 2.55)	0.022	3.00 (2.16 to 4.17	<0.0001

Chronic airflow obstruction: Analyses compared with those with no evidence of chronic airflow obstruction, that is, FEV_1_/FVC and FEV_1_/FEV_6_ greater than or equal to the LLN (n=25 383); Spirometric restriction: Analyses compared with those with no evidence of spirometric restriction, that is, FVC and FEV_6_ greater than or equal to the LLN (n=17 658). To calculate the LLN, we used sex-specific coefficients for age and height from reference equations for European Americans in the third US National Health and Nutrition Examination Survey.^20^ Dyspnoea was categorised as minimal/no breathlessness (0–1 on the mMRC dyspnoea scale) and significant breathlessness (≥2 on the mMRC dyspnoea scale); chronic cough, chronic phlegm and wheeze, categorised as yes/no by responses to the following questions: (1) ‘do you cough on most days for as much as 3 months each year?’; (2) ‘do you bring up phlegm on most days for as much 3 months each year?’; and (3) ‘have you had wheezing or whistling in the chest at any time in the last 12 months?’.

FEV_1_, forced expiratory volume in 1 s; FEV_6_, forced expiratory volume in 6 s; FVC, forced vital capacity; LLN, lower limit of normal; mMRC, modified Medical Research Council.

**Table 5:** Association of concordant and discordant measurements with physical and mental quality of life

	Physical quality of life (SF-12)		Mental quality of life (SF-12)	
	β (95% CI)	P value	β (95% CI)	P value
Chronic airflow obstruction				
FEV_1_/FVC and FEV_1_/FEV_6_<LLN (n=2013)	−2.87 (−3.48, −2.27)	<0.0001	−1.03 (−1.68, −0.39)	0.002
FEV_1_/FVC<LLN with FEV_1_/FEV_6_≥LLN (n=464)	−1.60 (−2.46, −0.74)	<0.0001	−0.44 (−1.38, 0.50)	0.357
FEV_1_/FVC≥LLN with FEV_1_/FEV_6_<LLN (n=283)	−1.91 (−3.20, −0.61)	0.004	−1.40 (−2.72, −0.10)	0.036
Spirometric restriction				
FVC and FEV_6_<LLN (n=7301)	−1.17 (−1.79, −0.54)	<0.0001	−0.21 (−0.58, 0.17)	0.285
FVC<LLN with FEV_6_≥LLN (n=701)	0.04 (−0.59, −0.67)	0.899	0.32 (−0.47, 1.10)	0.427
FVC≥LLN with FEV_6_<LLN (n=264)	−2.62 (−3.98, −1.27)	<0.0001	0.18 (−0.94, 1.30)	0.752

Physical and mental quality of life were measured using the SF-12 questionnaire. Negative regression coefficient indicates a reduction in SF-12 score. Chronic airflow obstruction: Analyses compared with those with no evidence of chronic airflow obstruction, that is, FEV_1_/FVC and FEV_1_/FEV_6_ greater than or equal to the LLN (n=20 977); Spirometric restriction: Analyses compared with those with no evidence of spirometric restriction, that is, FVC and FEV_6_ greater than or equal to the LLN (n=15 375). Analyses not including Benin (Sémé-Kpodji), Cameroon (Limbe), Jamaica, Kyrgyzstan (Chui), Kyrgyzstan (Naryn), Malaysia (Penang), Pakistan (Karachi) and Sri Lanka who measured quality of life using a different tool. To calculate the LLN, we used sex-specific coefficients for age and height from reference equations for European Americans in the third US National Health and Nutrition Examination Survey.^20^

FEV_1_, forced expiratory volume in 1 s; FEV_6_, forced expiratory volume in 6 s; FVC, forced vital capacity; LLN, lower limit of normal; SF-12, 12-item short form health survey.

18 study sites took part in follow-up between 29 January 2019 and 7 October 2021, with 12 502 eligible participants. At follow-up, 1155 participants had died, 3658 had migrated or were unreachable, 1237 refused to participate and 516 enrolled but never completed the core questionnaire. 5936 participants completed the core questionnaire at follow-up, from which 2066 participants were excluded due to not performing spirometry (n=855) or poor-quality spirometry (n=1211). A total of 3870 participants with a median (IQR) follow-up time of 8.3 years (6.1–11.0) were included in the longitudinal analyses.

The characteristics of study participants with good quality post-bronchodilator spirometry at baseline and follow-up are displayed in online supplemental eTable 4. Of those followed up, the baseline prevalence of FEV_1_/FEV_6_ ratio less than the LLN when the FEV_1_/FVC ratio was normal was 1% (37 of 2823) with 35% (13 of 37) having a post-bronchodilator FEV_1_/FVC ratio less than the LLN at follow-up. The baseline prevalence of having an FEV_6_ less than the LLN when the FVC was normal was 2% (33 of 1641) with 42% (14 of 33) having a post-bronchodilator FVC less than the LLN at follow-up. 12 of the 18 sites had instances of discordant measurements and were included as clusters in the multilevel analyses. Having an FEV_1_/FEV_6_ ratio less than the LLN when the FEV_1_/FVC ratio was normal at baseline was associated with a greater reduction in post-bronchodilator FEV_1_/FVC ratio (β: −8.45%, 95% CI, −11.27 to −5.64) and greater odds of having an FEV_1_/FVC ratio less than the LLN (OR: 8.80, 95% CI, 3.14 to 24.62) at follow-up, compared with those with both FEV_1_/FVC and FEV_1_/FEV_6_ ratios equal to or greater than the LLN (online supplemental eTable 5). Similarly, having an FEV_6_ less than the LLN when the FVC was normal at baseline was associated with a greater reduction in post-bronchodilator FVC (β: −0.25 L, 95% CI, −0.42 to –0.08) and greater odds of having an FVC less than the LLN (OR: 2.27, 95% CI, 1.14 to 4.52) at follow-up, compared with those with both FVC and FEV_6_ equal to or greater than the LLN (online supplemental eTable 6).

## Discussion

This study shows that the FEV_1_/FEV_6_ ratio and FEV_6_ may be used to identify chronic airflow obstruction and spirometric restriction. Our findings were similar between males and females and across world regions. Having discordant measurements was associated with lower lung function, a greater burden of respiratory symptoms, lower QoL and greater odds of progression to chronic airflow obstruction and spirometric restriction over time.

We found that the mean FVC was 50 mL to 150 mL larger than the FEV_6_ across world regions. The smallest difference was seen among those in the African, South-East Asian and Western Pacific regions, where lung capacity assessed by the FVC was the lowest and forced expiratory time the shortest. As a result, there was a smaller difference between the FEV_1_/FVC ratio and FEV_1_/FEV_6_ ratio in these regions compared with those of the European and American regions, where FVC was larger and forced expiratory time longer. This supports the recent update to the joint American Thoracic Society/European Respiratory Society spirometry (ATS/ERS) guidelines,^4^ which removed the requirement for forced expiration to be equal to or greater than 6 s to meet end of test criteria.

Post-bronchodilator FEV_1_/FEV_6_ ratio less than the LLN had excellent accuracy in identifying FEV_1_/FVC ratio less than the LLN. In a similar study, Bhatt and colleagues^7^ used data from over 10 000 participants of the COPDGene study and found slightly stronger agreement. However, they used fixed cut-offs that are no longer recommended by the ATS/ERS due to risk of misclassification,^4 24^ meaning our results are not directly comparable. Similarly, Rosa and colleagues^12^ found excellent agreement between post-bronchodilator FEV_1_/FVC and FEV_1_/FEV_6_ ratios when using the LLN to define abnormality, in 1000 participants of the population-based Proyecto LatinoAmericano de Investigación en Obstrucción Pulmonar (PLATINO) study. Our results add further evidence that in general populations, the post-bronchodilator FEV_1_/FEV_6_ ratio can be used as a surrogate for the FEV_1_/FVC ratio. We did see some variation in accuracy and agreement across WHO regions. The regions with the strongest agreement tended to be those with the smaller and more comparable mean FEV_6_ and FVC measurements, lower prevalence of chronic airflow obstruction and subsequently a shorter forced expiratory time. Suggesting that factors that increase expiratory time, as seen in the European and American regions, can impact the strength of agreement.^16^

The accuracy and agreement for the FEV_6_ less than the LLN to identify FVC less than the LLN was excellent. There was also minimal variation in agreement across world regions. There are no population-based studies for direct comparison. However, Vandevoorde and colleagues^14^ evaluated the accuracy of the FEV_6_ less than the LLN to identify spirometric restriction in 11 676 patients referred for lung function testing at a Brussels hospital. They found that the FEV_6_ had a sensitivity of 82.7% and specificity of 99.6% for identifying spirometric restriction, similar to the 84.6% and 98.6% we found for the European sites of the BOLD study. In combination, our results suggest that when using the LLN, the FEV_6_ is an acceptable surrogate for the FVC in both high-risk patient and general populations.

We found that 1% of BOLD study participants had an FEV_1_/FEV_6_ ratio less than the LLN when the FEV_1_/FVC ratio was normal. This is similar to both Bhatt and colleagues^7^ and Rosa and colleagues,^12^ who found that 1% and 2% of study populations, respectively, had discordant reductions in FEV_1_/FEV_6_ ratio. Other studies found discordance of up to 7%; however, there was great variation in study designs, including hospital-based populations, pre-bronchodilator measurements and fixed cut-offs.^5 6 8 14 15^ In our study, those with a discordant reduction in FEV_1_/FEV_6_ ratio had lower lung function, greater odds of being symptomatic and lower physical QoL compared with those with normal lung function. Which together with the finding that they are also more likely to progress to chronic airflow obstruction over time, supports previous research showing that discordant reductions in FEV_1_/FEV_6_ ratio reflect a genuine physiological limitation.^7 25^ It is therefore not surprising that those with a discordant reduction in FEV_1_/FEV_6_ ratio had an FEV_1_/FVC ratio close to the LLN, demonstrating the importance of considering other indicators such as smoking history and respiratory symptoms when interpreting borderline spirometry results.^26^

For spirometric restriction, we found that 1% had a discordant reduction in FEV_6_. This is very similar to Rosa and colleagues^12^ and Vandevoorde and colleagues,^14^ who found that 1% and 2%, respectively, had discordant measurements. We found that this was associated with lower lung function, greater odds of all respiratory symptoms, lower physical QoL and greater odds of progressing to spirometric restriction over time. Of particular interest was the finding that the mean FEV_1_/FVC ratio in this population was 59%. This suggests that some people with a discordant reduction in FEV_6_ also have obstruction, which increases expiratory time and potentially explains the discordance seen.^25^

Our study has several strengths. First, its large sample size and population-based design make the results transferable to general populations. Spirometry was conducted by trained and certified technicians, and lung function data was quality assured centrally. A further strength is the administration of standardised questionnaires in local languages. Our study also has some limitations. We did not use the Global Lung Initiative reference equations as they do not provide equations for the FEV_1_/FEV_6_ ratio and FEV_6_,^27^ which restricted our ability to use multiethnic reference values. However, race-correction has been shown not to affect the prevalence estimates of airflow obstruction in the NHANES study population.^28^ While for spirometric restriction, there is evidence that race-correction of the FVC LLN misclassifies individuals as normal who have underlying disease and increased risk of mortality.^29^ Furthermore, we do not have data on whether an expiratory plateau was achieved according to the most up-to-date quality criteria,^4^ as the BOLD study data collection took place before these criteria were published. The longitudinal component of this study was impacted by significant loss to follow-up caused by the COVID-19 pandemic. Although we attempted to account for this by using inverse probability weights, it is possible that those present at follow-up are not entirely representative of the general population.

In conclusion, we have shown that there is strong agreement between the FVC and FEV_6_ in the identification of chronic airflow obstruction and spirometric restriction, sufficient for their use to be interchangeable in most circumstances. However, relying on either method alone can result in a small number being misclassified as normal when symptomatic, possibly indicating underlying disease.

## Supplementary material

10.1136/bmjresp-2024-002355 online supplemental file 1

## Data Availability

Data are available upon reasonable request.
