# Supplementary material for: Concordance between FVC and FEV6 for identifying chronic airflow obstruction and spirometric restriction in the Burden of Obstructive Lung Disease (BOLD) study
Source: BMJ Open Respir Res. 2025 Jul 13;12(1):e002355. doi: 10.1136/bmjresp-2024-002355 (PMC12258291; doi:10.1136/bmjresp-2024-002355)
Supplement: online supplemental file 1 [file bmjresp-12-1-s001.docx]

**Contents**

[**eTable 1. Characteristics of the BOLD study population with good quality post-bronchodilator spirometry.** 2](#_Toc195267146)

[**eTable 2. Ability of pre-bronchodilator FEV_1_/FEV_6_ and FEV_6_ less than the LLN to identify airflow obstruction and spirometric restriction defined using FEV_1_/FVC and FVC less than the LLN.** 3](#_Toc195267147)

[**eTable 3. Ability of FEV_6_ to identify spirometric restriction defined using FVC in those with normal FEV_1/_FVC and FEV_1_/FEV_6_ ratios.** 4](#_Toc195267148)

[**eTable 4. Characteristics of BOLD study participants with good quality post-bronchodilator spirometry at baseline and follow-up.** 5](#_Toc195267149)

[**eTable 5. Association between baseline discordant FEV_1_/FEV_6_ less than the LLN and chronic airflow obstruction at follow-up.** 6](#_Toc195267150)

[**eTable 6. Association between baseline discordant FEV_6_ less than the LLN and spirometric restriction at follow-up.** 6](#_Toc195267151)

[**References** 7](#_Toc195267152)

## **eTable 1. Characteristics of the BOLD study population with good quality post-bronchodilator spirometry.**

| **BOLD Centre** | ***n*** | **Females**  ***n* (%)** | **Age, yr**  **Mean (SD)** | **BMI**  **Mean (SD)** | **Ever smoke**  ***n* (%)** | **FEV_1_/FVC (%)**  **Mean (SD)** | **FEV_1_/FEV_6_ (%)**  **Mean (SD)** | **FVC (L)**  **Mean (SD)** | **FEV_6_ (L)**  **Mean (SD)** |
| --- | --- | --- | --- | --- | --- | --- | --- | --- | --- |
| Albania (Tirana) | 939 | 472 (50%) | 54.6 (10.8) | 28.0 (4.7) | 348 (37%) | 78.4 (9.0) | 80.7 (7.1) | 3.6 (0.9) | 3.5 (0.9) |
| Algeria (Annaba) | 890 | 448 (50%) | 52.5 (9.9) | 28.3 (5.7) | 341 (38%) | 78.6 (7.3) | 80.1 (6.6) | 3.4 (0.9) | 3.4 (0.9) |
| Australia (Sydney) | 541 | 276 (51%) | 59.9 (12.4) | 28.0 (5.2) | 275 (51%) | 76.4 (8.9) | 79.4 (7.1) | 3.6 (1.0) | 3.4 (1.0) |
| Austria (Salzburg) | 1258 | 573 (46%) | 57.7 (11.4) | 26.4 (4.2) | 663 (53%) | 74.3 (8.6) | 77.7 (6.9) | 4.0 (1.0) | 3.8 (1.0) |
| Benin (Sémé-Kpodji) | 694 | 394 (57%) | 51.6 (9.8) | 26.4 (5.6) | 13 (2%) | 79.3 (7.1) | 80.9 (6.1) | 2.8 (0.7) | 2.7 (0.7) |
| Cameroon (Limbe) | 287 | 116 (40%) | 52.2 (9.4) | 26.6 (5.4) | 64 (22%) | 80.6 (7.0) | 81.6 (6.4) | 3.0 (0.8) | 2.9 (0.7) |
| Canada (Vancouver) | 826 | 483 (58%) | 56.1 (11.9) | 26.7 (5.2) | 434 (52%) | 76.0 (8.9) | 79.5 (6.5) | 3.9 (1.1) | 3.8 (1.1) |
| China (Guangzhou) | 473 | 237 (50%) | 54.1 (10.7) | 23.3 (3.3) | 207 (44%) | 77.9 (7.7) | 79.4 (6.9) | 3.1 (0.8) | 3.0 (0.8) |
| England (London) | 677 | 354 (52%) | 58.2 (11.5) | 27.1 (5.0) | 419 (62%) | 75.0 (9.2) | 78.2 (7.1) | 3.7 (1.0) | 3.5 (1.0) |
| Estonia (Tartu) | 613 | 305 (50%) | 60.9 (12.0) | 28.5 (5.3) | 290 (47%) | 77.2 (7.8) | 79.5 (6.3) | 3.8 (1.1) | 3.7 (1.0) |
| Germany (Hannover) | 683 | 334 (49%) | 58.1 (11.0) | 27.3 (4.6) | 409 (60%) | 76.2 (7.9) | 78.9 (6.6) | 3.9 (1.0) | 3.8 (1.0) |
| Iceland (Reykjavik) | 757 | 354 (47%) | 56.4 (11.7) | 27.9 (4.9) | 462 (61%) | 76.1 (8.5) | 79.1 (6.7) | 4.0 (1.0) | 3.9 (1.0) |
| India (Mumbai) | 440 | 165 (38%) | 51.0 (8.9) | 23.8 (4.0) | 43 (10%) | 79.1 (7.5) | 79.7 (7.1) | 2.8 (0.7) | 2.8 (0.8) |
| India (Mysore) | 601 | 345 (57%) | 46.7 (7.2) | 24.7 (3.8) | 48 (8%) | 79.5 (7.4) | 80.7 (6.7) | 2.6 (0.7) | 2.5 (0.7) |
| India (Pune) | 782 | 318 (41%) | 52.3 (9.8) | 22.1 (3.9) | 95 (12%) | 79.5 (8.1) | 80.9 (7.5) | 2.8 (0.7) | 2.7 (0.7) |
| India (Kashmir) | 757 | 343 (45%) | 51.4 (10.4) | 22.4 (3.6) | 95 (13%) | 76.4 (10.6) | 78.6 (8.8) | 3.3 (0.9) | 3.1 (0.9) |
| Jamaica | 578 | 335 (58%) | 55.9 (11.6) | 27.5 (6.6) | 173 (30%) | 78.4 (9.2) | 80.5 (7.6) | 3.0 (0.8) | 2.9 (0.8) |
| Kyrgyzstan (Chui) | 858 | 588 (69%) | 53.0 (8.8) | 28.5 (5.6) | 253 (29%) | 77.2 (8.2) | 79.6 (6.6) | 3.4 (0.9) | 3.3 (0.8) |
| Kyrgyzstan (Naryn) | 820 | 505 (62%) | 53.3 (10.0) | 27.0 (5.0) | 203 (25%) | 77.8 (7.3) | 80.3 (5.7) | 3.5 (0.9) | 3.4 (0.9) |
| Malawi (Blantyre) | 401 | 241 (60%) | 52.3 (9.9) | 25.0 (5.4) | 53 (13%) | 78.2 (7.8) | 80.7 (6.4) | 3.0 (0.7) | 2.9 (0.7) |
| Malawi (Chikwawa) | 432 | 211 (49%) | 53.8 (10.5) | 21.8 (3.9) | 130 (30%) | 76.3 (9.1) | 79.1 (7.4) | 3.1 (0.7) | 3.0 (0.7) |
| Malaysia (Penang) | 663 | 323 (49%) | 54.5 (9.5) | 26.1 (4.5) | 168 (25%) | 81.0 (6.8) | 82.2 (5.9) | 2.7 (0.7) | 2.6 (0.7) |
| Morocco (Fes) | 768 | 414 (54%) | 55.1 (10.3) | 27.9 (5.3) | 210 (27%) | 78.1 (8.3) | 80.3 (0.9) | 3.3 (0.9) | 3.2 (0.8) |
| Netherlands (Maastricht) | 590 | 290 (49%) | 57.5 (10.7) | 27.4 (4.5) | 385 (65%) | 74.6 (10.0) | 78.2 (7.6) | 4.0 (1.0) | 3.8 (1.0) |
| Nigeria (Ife) | 883 | 538 (61%) | 55.4 (12.0) | 25.3 (5.4) | 94 (11%) | 78.5 (8.4) | 80.8 (6.6) | 2.7 (0.7) | 2.6 (0.7) |
| Norway (Bergen) | 658 | 334 (51%) | 59.8 (12.6) | 26.5 (4.3) | 413 (63%) | 74.9 (8.8) | 78.2 (7.0) | 3.9 (1.1) | 3.7 (1.0) |
| Pakistan (Karachi) | 607 | 339 (56%) | 51.6 (9.6) | 26.5 (5.5) | 134 (22%) | 80.0 (9.7) | 81.8 (8.2) | 2.5 (0.8) | 2.5 (0.7) |
| Philippines (Manila) | 893 | 515 (58%) | 52.3 (10.2) | 24.9 (4.7) | 472 (53%) | 79.0 (8.9) | 80.6 (7.7) | 2.6 (0.7) | 2.5 (0.7) |
| Philippines (Nampicuan-Talugtug) | 722 | 366 (51%) | 54.1 (10.5) | 21.5 (3.9) | 380 (53%) | 77.0 (10.6) | 78.7 (9.3) | 2.7 (0.8) | 2.7 (0.8) |
| Poland (Krakow) | 526 | 260 (49%) | 55.7 (11.5) | 27.7 (4.6) | 323 (61%) | 75.1 (9.2) | 78.1 (7.4) | 3.8 (1.0) | 3.7 (1.0) |
| Portugal (Lisbon) | 710 | 379 (53%) | 63.4 (11.3) | 28.2 (4.6) | 284 (40%) | 75.8 (9.0) | 79.3 (6.6) | 3.2 (0.9) | 3.0 (0.9) |
| Saudi Arabia (Riyadh) | 700 | 325 (46%) | 50.3 (7.7) | 31.2 (6.0) | 171 (24%) | 82.6 (6.0) | 83.4 (5.4) | 3.0 (0.8) | 3.0 (0.8) |
| South Africa (Uitsig and Ravensmead) | 843 | 531 (63%) | 54.2 (10.5) | 27.9 (7.5) | 570 (68%) | 75.7 (11.2) | 78.1 (9.4) | 2.9 (0.8) | 2.8 (0.8) |
| Sri Lanka | 1023 | 566 (55%) | 53.7 (9.5) | 24.2 (4.6) | 209 (20%) | 79.7 (8.7) | 80.7 (7.9) | 2.3 (0.6) | 2.3 (0.6) |
| Sudan (Gezeira) | 581 | 281 (48%) | 53.8 (10.1) | 27.3 (17.3) | 132 (23%) | 80.1 (7.2) | 81.3 (6.6) | 3.0 (0.8) | 2.9 (0.8) |
| Sudan (Khartoum) | 516 | 210 (41%) | 54.0 (10.4) | 26.4 (6.4) | 120 (23%) | 77.9 (8.4) | 80.4 (6.8) | 2.9 (0.8) | 2.8(0.7) |
| Sweden (Uppsala) | 547 | 264 (48%) | 58.4 (10.9) | 27.0 (4.4) | 314 (57%) | 76.3 (8.0) | 79.1 (6.4) | 4.0 (1.1) | 4.8 (1.0) |
| Trinidad & Tobago (Port of Spain) | 1092 | 657 (60%) | 54.1 (10.8) | 29.1 (10.0) | 297 (27%) | 79.6 (7.6) | 81.7 (6.3) | 2.7 (0.8) | 2.6 (0.8) |
| Tunisia (Sousse) | 661 | 352 (53%) | 53.0 (9.1) | 29.2 (5.6) | 263 (40%) | 80.0 (7.5) | 81.3 (6.5) | 3.4 (0.9) | 3.3 (0.9) |
| Turkey (Adana) | 806 | 417 (52%) | 53.6 (10.4) | 29.6 (5.3) | 441 (55%) | 75.8 (8.7) | 78.6 (7.0) | 3.5 (0.9) | 3.3 (0.9) |
| USA (Lexington) | 508 | 302 (59%) | 56.6 (9.9) | 30.8 (6.8) | 306 (60%) | 76.2 (9.4) | 78.5 (8.0) | 3.4 (1.0) | 3.3 (1.0) |
| Overall | 28604 | 15060 (53%) | 54.7 (10.9) | 26.7 (6.2) | 10701 (37%) | 77.8 (8.7) | 79.9 (7.2) | 3.2 (1.0) | 3.1 (0.9) |

*Categorical variables presented as number (%), continuous variables as mean (SD). BMI: Body mass index; FEV_1_: Forced expiratory volume in one second; FVC: Forced vital capacity; FEV_6_: Forced expiratory volume in six seconds; L: litres*.

## **eTable 2.** **Ability of pre-bronchodilator FEV_1_/FEV_6_ and FEV_6_ less than the LLN to identify airflow obstruction and spirometric restriction defined using FEV_1_/FVC and FVC less than the LLN.**

| **Airflow obstruction** | | | | | | |
| --- | --- | --- | --- | --- | --- | --- |
|  | ***n*** | **Level of agreement %** | **Sensitivity**  **%** | **Specificity**  **%** | **AUC**  **(95% CI)** | **Kappa coefficient (SE)** |
| **Overall** | 27158 | 95.50 | 79.50 | 98.16 | 0.89 (0.88-0.90) | 0.81 (0.01) |
| **Male** | 12851 | 95.40 | 81.24 | 97.99 | 0.90 (0.89-0.91) | 0.82 (0.01) |
| **Female** | 13307 | 95.60 | 77.68 | 98.31 | 0.88 (0.87-0.89) | 0.80 (0.01) |
| **Spirometric restriction** | | | | | | |
| **WHO region** | ***n*** | **Level of agreement %** | **Sensitivity**  **%** | **Specificity**  **%** | **AUC**  **(95% CI)** | **Kappa coefficient (SE)** |
| **Overall** | 27158 | 95.73 | 93.17 | 97.27 | 0.95 (0.94-0.96) | 0.91 (0.01) |
| **Male** | 12851 | 95.17 | 91.78 | 97.13 | 0.95 (0.94-0.95) | 0.89 (0.01) |
| **Female** | 13307 | 96.23 | 94.36 | 97.41 | 0.96 (0.95-0.96) | 0.92 (0.01) |

*Level of agreement classified according to Cohen 1960^1^, 0·01-0·20= none to minimal, 0·21-0·40= slight, 0·41-0·60= moderate, 0·61- 0·80=substantial, 0·81-1·0= almost perfect. AUC: Area under the curve; 95%CI: 95% confidence interval; SE: Standard error. FEV1: Forced expiratory volume in one second; FEV6: Forced expiratory volume in 6 seconds; FVC: Forced vital capacity. LLN: Lower limit of normal. Spirometric condition identified if a result is less than the LLN.* *To calculate the LLN, we used sex-specific coefficients for age and height from reference equations for European Americans in the third US National Health and Nutrition Examination Survey (NHANES)^2^*

## **eTable 3. Ability of FEV_6_ to identify spirometric restriction defined using FVC in those with normal FEV_1/_FVC and FEV_1_/FEV_6_ ratios.**

| **Spirometric restriction** | | | | | | |
| --- | --- | --- | --- | --- | --- | --- |
| **WHO region** | ***n*** | **Level of agreement %** | **Sensitivity**  **%** | **Specificity**  **%** | **AUC**  **(95% CI)** | **Kappa coefficient (SE)** |
| **Overall** | 28604 | 95.33 | 88.34 | 98.84 | 0.94 (0.93-0.94) | 0.89 (0.01) |
| **Male** | 13544 | 94.78 | 85.95 | 98.82 | 0.92 (0.92-0.93) | 0.88 (0.01) |
| **Female** | 15060 | 95.86 | 90.29 | 98.85 | 0.95 (0.95-0.96) | 0.91 (0.01) |
| **WHO region** |  |  |  |  |  |  |
| African | 4430 | 94.15 | 90.24 | 97.62 | 0.94 (0.93-0.95) | 0.88 (0.02) |
| Americas | 3004 | 95.44 | 91.71 | 97.98 | 0.95 (0.94-0.96) | 0.91 (0.02) |
| Eastern Mediterranean | 3833 | 93.84 | 88.19 | 98.20 | 0.93 (0.92-0.94) | 0.87 (0.02) |
| European | 10442 | 97.49 | 80.30 | 99.34 | 0.90 (0.88-0.91) | 0.85 (0.01) |
| South-East Asia | 3603 | 93.37 | 89.64 | 98.72 | 0.94(0.93-0.95) | 0.87 (0.02) |
| Western Pacific | 3292 | 93.86 | 86.66 | 99.46 | 0.93 (0.92-0.94) | 0.87 (0.02) |

*Level of agreement classified according to Cohen 1960^1^, 0·01-0·20= none to minimal, 0·21-0·40= slight, 0·41-0·60= moderate, 0·61- 0·80=substantial, 0·81-1·0= almost perfect. WHO: World Health Organisation; AUC: Area under the curve; 95%CI: 95% confidence interval; SE: Standard error. FEV1: Forced expiratory volume in one second; FEV6: Forced expiratory volume in 6 seconds; FVC: Forced vital capacity. LLN: Lower limit of normal. Chronic airflow obstruction defined if the FEV_1_/FVC (gold standard) or FEV_1_/FEV_6_ were less than the lower limit of normal (LLN). Spirometric restriction defined if the FVC was less than the LLN with FEV_1_/FVC equal to or greater than the LLN (gold standard) or the FEV_6_ was less than the LLN with FEV_1_/FEV_6_ equal to or greater than the LLN. To calculate the LLN, we used sex-specific coefficients for age and height from reference equations for European Americans in the third US National Health and Nutrition Examination Survey (NHANES)^1^.*

| **Total n = 3870** | **Participants present at follow-up**  ***n*** | **Females**  ***n* (%)** | **Age at baseline, yrs**  ***Mean (SD)*** | **Ever smoke at baseline**  ***n (%)*** | **FEV_1_/FEV_6_ <LLN with FEV_1_/FVC ≥LLN at baseline**  ***n (%)*** | **FEV_1_/FEV_6_ <LLN with FEV_1_/FVC ≥LLN at baseline with FEV_1_/FVC <LLN at follow-up**  ***n (%)*** | **FEV_6_ <LLN with FVC ≥LLN at baseline**  ***n (%)*** | **FEV_6_ <LLN with FVC ≥LLN at baseline with FVC <LLN at follow-up**  ***n (%)*** | **Follow-up time**  ***Median (IQR)*** |
| --- | --- | --- | --- | --- | --- | --- | --- | --- | --- |
| Benin (Sémé-Kpodji) | 102 | 56 (55%) | 50.7 (7.9) | 1 (1%) | 1 (1%) | 0 (0%) | 1 (1%) | 1 (100%) | 7.0 (6.9-7.1) |
| Estonia (Tartu) | 192 | 98 (51%) | 58.7 (10.3) | 83 (43%) | 0 (0%) | 0 (0%) | 0 (0%) | 0 (0%) | 10.9 (10.4-11.4) |
| Iceland (Reykjavik) | 270 | 133 (49%) | 50.8 (8.1) | 154 (57%) | 0 (0%) | 0 (0%) | 2 (1%) | 1 (50%) | 14.5 (14.3-15.0) |
| India (Kashmir) | 55 | 20 (36%) | 53.6 (10.5) | 5 (9%) | 3 (5%) | 2 (67%) | 1 (1%) | 0 (0%) | 8.5 (8.4-8.6) |
| India (Mysore) | 413 | 249 (60%) | 45.9 (6.5) | 25 (6%) | 4 (1%) | 1 (25%) | 1 (0%) | 0 (0%) | 7.2 (6.7-8.0) |
| India (Pune) | 466 | 205 (44%) | 50.4 (8.4) | 41 (9%) | 2 (0.5%) | 1 (50%) | 0 (0%) | 0 (0%) | 10.9 (10.7-11.1) |
| Jamaica (Kingston) | 26 | 11 (42%) | 52.5 (8.2) | 7 (27%) | 0 (0%) | 0 (0%) | 0 (0%) | 0 (0%) | 5.5 (5.3-5.6) |
| Kyrgyzstan (Chui) | 287 | 206 (72%) | 51.2 (7.5) | 72 (25%) | 1 (0%) | 0 (0%) | 3 (1%) | 2 (67%) | 6.1 (6.0-6.2) |
| Kyrgyzstan (Naryn) | 295 | 185 (63%) | 50.7 (7.6) | 65 (22%) | 3 (1%) | 1 (33%) | 3 (1%) | 0 (0%) | 6.1 (6.0-6.2) |
| Malawi (Chikwawa) | 267 | 147 (55%) | 53.1 (10.0) | 73 (27%) | 4 (2%) | 3 (75%) | 3 (1%) | 2 (67%) | 4.8 (4.4-5.0) |
| Morocco (Fes) | 18 | 5 (28%) | 49.8 (5.3) | 9 (50%) | 0 (0%) | 0 (0%) | 0 (0%) | 0 (0%) | 10.6 (10.2-10.9) |
| Nigeria (Ife) | 366 | 263 (72%) | 54.7 (11.4) | 28 (8%) | 4 (1%) | 0 (0%) | 10 (3%) | 5 (50%) | 8.3 (8.2-8.4) |
| Norway (Bergen) | 237 | 120 (51%) | 53.4 (8.4) | 156 (66%) | 0 (0%) | 0 (0%) | 0 (0%) | 0 (0%) | 13.9 (3.3-14.7) |
| Pakistan (Karachi) | 207 | 122 (59%) | 49.9 (8.5) | 43 (21%) | 5 (2%) | 1 (20%) | 4 (2%) | 1 (25%) | 4.4 (4.0-4.7) |
| Philippines (Nampicuan-Talugtug) | 276 | 153 (55%) | 51.1 (8.5) | 125 (45%) | 3 (1%) | 2 (67%) | 2 (1%) | 1 (50%) | 10.7 (10.6-11.0) |
| Sudan (Khartoum) | 35 | 14 (40%) | 51.2 (9.6) | 14 (40%) | 0 (0%) | 0 (0%) | 0 (0%) | 0 (0%) | 7.4 (7.3-7.5) |
| Sweden (Uppsala) | 202 | 92 (45%) | 54.7 (8.1) | 117 (58%) | 2 (1%) | 0 (0%) | 1 (0%) | 0 (0%) | 13.3 (13.0-13.7) |
| Tunisia (Sousse) | 156 | 87 (56%) | 52.6 (8.6) | 52 (33%) | 5 (3%) | 3 (60%) | 2 (1%) | 1 (50%) | 10.6 (10.4-10.8) |

## **eTable 4. Characteristics of BOLD study participants with good quality post-bronchodilator spirometry at baseline and follow-up.**

*SD: Standard deviation; IQR: Interquartile range; At baseline, discordant FEV_1_/FEV_6_ was identified if the post-bronchodilator forced expiratory volume in 1 second as a ratio of the forced expiratory volume in 6 seconds (FEV_1_/FEV_6_) was less than the LLN with a post-bronchodilator forced expiratory volume in 1 second as a ratio of the forced vital capacity (FEV_1_/FVC) equal to or above the LLN. At follow-up, chronic airflow obstruction was identified if the post-bronchodilator (200mcg salbutamol) FEV_1_/FVC ratio was less than the LLN. At baseline, discordant FEV_6_ was identified if the post-bronchodilator forced expiratory volume in 6 seconds (FEV_6_) was less than the LLN with a post-bronchodilator forced vital capacity (FVC) equal to or above the LLN. Spirometric restriction was diagnosed if the post-bronchodilator (200mcg salbutamol) FVC was less than the LLN at follow-up. LLN calculated using reference equations from the NHANES III study population^1^.*

## **eTable 5. Association between baseline discordant FEV_1_/FEV_6_ less than the LLN and chronic airflow obstruction at follow-up.**

| **Total**  ***n*** | **Discordant**  **FEV_1_/FEV_6_ <LLN**  ***n (%)*** | **Chronic airflow obstruction**  **(follow-up)**  ***n (%)*** | **OR**  **(95%CI)** | **p-value** | **β coefficient**  **(95%CI)^*^** | **p-value** |
| --- | --- | --- | --- | --- | --- | --- |
| 2823 | 37 (1%) | 14 (35%) | 8.80 (3.14-24.62) | <0.0001 | -8.45 (-11.27, -5.64) | <0.0001 |

*Linear associations* *between* *discordant FEV_1_/FEV_6_ less than the lower limit of normal (LLN) at baseline and follow-up post-bronchodilator FEV_1_/FVC ratio were estimated using a mixed effects linear regression model *Negative regression coefficient indicates a reduction in FEV_1_/FVC ratio (i.e., worsened lung function). Associations between discordant FEV_1_/FEV_6_ less than the LLN at baseline and progression to chronic airflow obstruction (CAO) were estimated using mixed effects logistic regression models. Models were adjusted for sex, age, BMI, follow-up time, smoking status, and smoking pack years. As we expected associations to vary by study site, we fitted a random slope model to average the associations across BOLD study sites. The associations were averaged across 12 clusters, excluding Sudan, Norway, Morocco, Jamaica, Iceland, and Estonia, who had no cases of discordant spirometry at baseline. Discordant FEV_1_/FEV_6_ was identified if the post-bronchodilator forced expiratory volume in 1 second as a ratio of the forced expiratory volume in 6 seconds (FEV_1_/FEV_6_) was less than the LLN at baseline with a post-bronchodilator forced expiratory volume in 1 second as a ratio of the forced vital capacity (FEV_1_/FVC) equal to or above the LLN. CAO was diagnosed if the post-bronchodilator (200mcg salbutamol) FEV_1_/FVC was less than the LLN at follow-up. LLN calculated using reference equations from the NHANES III study population^1^. Total n= those within the 12 clusters, without chronic airflow obstruction at baseline, who had a measurement for post-bronchodilator FEV_1_/FVC at follow-up_._*

## **eTable 6. Association between baseline discordant FEV_6_ less than the LLN and spirometric restriction at follow-up.**

| **Total**  ***n*** | **Discordant**  **FEV_6_ <LLN**  ***n (%)*** | **Spirometric**  **restriction**  **(follow-up)**  ***n (%)*** | **OR**  **(95%CI)** | **p-value** | **β coefficient**  **(95%CI)^*^** | **p-value** |
| --- | --- | --- | --- | --- | --- | --- |
| 1641 | 33 (2%) | 14 (42%) | 2.27 (1.14-4.52) | 0.020 | -0.25 (-0.42, -0.08) | 0.005 |

*Linear associations between discordant FEV_6_ less than the lower limit of normal (LLN) at baseline and follow-up post-bronchodilator FVC ratio were estimated using a mixed effects linear regression model *Negative regression coefficient indicates a reduction in FVC ratio (i.e., worsened lung function). Associations between discordant FEV_6_ less than the LLN at baseline and progression to spirometric restriction were estimated using mixed effects logistic regression models. Models were adjusted for sex, age, BMI, follow-up time, smoking status, and smoking pack years. As we expected associations to vary by study site, we fitted a random slope model to average the associations across BOLD study sites. The associations were averaged across 12 clusters, excluding Sudan, Norway, Morocco, Jamaica, Estonia, and Pune (India), who had no cases of discordant spirometry at baseline. Discordant FEV_6_ was identified if the post-bronchodilator forced expiratory volume in 6 seconds (FEV_6_) was less than the LLN at baseline with a post-bronchodilator forced vital capacity (FVC) equal to or above the LLN. Spirometric restriction was diagnosed if the post-bronchodilator (200mcg salbutamol) FVC was less than the LLN at follow-up. LLN calculated using reference equations from the NHANES III study population^1^. Total n= those within the 12 clusters, without spirometric restriction at baseline, who had a measurement for FEV_6_, and were present at follow-up_._*

## **References**

1. Hankinson JL, Odencrantz JR, Fedan KB. Spirometric reference values from a sample of the general U.S. population. Am J Respir Crit Care Med 1999; 159(1): 179-87.

2. Cohen J. A coefficient of agreement for nominal scales. *Educational and Psychological Measurement* 1960; **20**: 37-46.

3. Hankinson JL, Odencrantz JR, Fedan KB. Spirometric reference values from a sample of the general U.S. population. *Am J Respir Crit Care Med* 1999; **159**(1): 179-87.
